# College Football Overtime Outcomes: Implications for In-Game Decision-Making

**DOI:** 10.3389/frai.2020.00061

**Published:** 2020-08-26

**Authors:** Rick L. Wilson

**Affiliations:** Department of Management Science and Information Systems, Spears School of Business, Oklahoma State University, Stillwater, OK, United States

**Keywords:** sports, football, analytics, machine learning, decision making

## Abstract

The use of AI and machine learning in sports is increasingly prevalent, including their use for in-game strategy and tactics. This paper reports on the use of machine learning techniques, applying it to analysis of U.S. Division I-A College Football overtime games. The present overtime rules for tie games in Division I-A college football was adopted in 1996. Previous research (Rosen and Wilson, 2007) found little to suggest that the predominantly used strategy of going on defense first was advantageous. Over the past decade, even with significant transformation of new offensive and defensive strategies, college football coaches still opt for the same conventional wisdom strategy. In revisiting this analysis of overtime games using both logistic regression and inductive learning/decision tree analysis, the study validates there remains no advantage to the defense first strategy in overtime. The study found evidence that point spread (as an indicator of team strength) and red zone offense performance of both teams were useful to predict game results. Additionally, by altering the decision-making “frame,” specific scenarios are illustrated where a coach can use these machine learning discovered relationships to influence end-of-regulation game decisions that may increase their likelihood of winning whether in regulation time or in overtime.

## Introduction

The practical and widespread use of sports analytics continues to increase across the entire industry. Artificial intelligence and machine learning techniques continue to be adopted in areas involving scouting and recruiting, training and performance analysis, revenue management strategies, and broadcasting and streaming decisions, in addition to strategic and tactical decisions “on the field” (Rein and Memmert, 2016; Joshi, 2019). This use of analytics transcends different sports, including those who have a long history of many statistics (such as baseball) to those which are more nascent in the use of big data (e.g., de Leeuw et al., 2018).

This paper revisits one such “on the field” decision—participation in overtime games in American college football. In revisiting this unique phenomenon in Division I-A college football games, the research uses additional data and a different decision lens in examining team attribute relationships to overtime game success. Machine learning approaches (regression and decision tree algorithms) are applied to historical game data, resulting in additional insight into overtime game outcomes that could also influence end-of-game decisions.

## Literature/Application Review

Note: This section assumes working basic knowledge of rules and scoring of American college football.

Until the 1996 regular season, major college football (Division I-A teams) in the U.S. allowed games to end in a tie. While there have been some monumentally famous and exciting tie games in college football's history, a tie game typically leaves little satisfaction to either teams or their fans.

Starting officially during the 1996 regular season, Division I-A College Football adopted what was known as the “Kansas Plan” to determine a winner for games that ended in a tie after the normal four quarters of play. A coin toss was held prior to the start of the overtime period(s), and the team that won the coin toss could choose to either have the ball first or go on defense first. Not surprisingly, the coaching conventional wisdom was that there is an advantage to playing defense first (much like batting last in baseball or cricket), so there was little or no variation at this “decision” stage—practically 100% of all coin toss winners elected to go on defense first.

Each team receives one possession in the overtime period, starting on the opponent 25-yard line (25 yards away from a touchdown), playing with the same game rules as in regulation and attempt to score either a touchdown or a field goal. It is worth noting that after a touchdown, teams retain the choice to either kick the extra point (a highly likely occurrence for one point) or try to score from the three-yard line for two points (the so-called two-point conversion, which is successfully <50% of the time).

If the score remains tied after the first overtime, the teams repeat the process but switch order of possession—the team that was on defense first in the first overtime has the ball first in the second overtime, and vice versa.

The teams continue playing until there is a winner, with the teams changing “the order” with each successive period until finally one team outscores the other. Thus, if a third period was needed, then the order of defense first/offense first would revert back to the original overtime period.

Due to unintended lengthy overtime games, rules have been modified twice to try to shorten the number of overtimes needed to declare a victor. In 1997, teams were mandated to go for two points after touchdowns from the 3rd overtime period and beyond. Most recently, in 2019, a rule change requires that teams rotate two-point conversion tries beginning in the fifth overtime, eliminating possessions at the 25-yard line. In these rare cases, each offense will have just one chance to convert the two-point try before the other team gains possession.

Oftentimes, the college football rules are contrasted with the rules used by the National Football League (NFL) for overtime, which are still widely criticized. In the NFL, a team can win the coin toss, elect to get the ball, then score a touchdown and win the game without the other team having a chance to score. The NFL rules appear to be unfair to the team that loses the coin toss. As this research will again show, the coin toss in college football overtime has virtually no tangible effect on game outcome, and thus universally, the college football overtime process is viewed to be “fair”.

Rosen and Wilson (2007) looked at the set of overtime games that had occurred since the beginning of the 1996 season (through 2005) and assessed whether the belief of the “large advantage” of going on defense first (held by coaches, fans, the press, etc.) was valid. Using discriminant analysis and decision tree analysis, little evidence was found indicating any advantage to being on defense first, and the paper hypothesized some scenarios where it could be advantageous for teams to choose to go offense first. Thus, Rosen and Wilson focused on what actions should be taken *after the* coin flip.

The present study uses some of the same elements of the previous study but utilizes a different decision lens framework. Using the most recent 7 years of overtime game data (2013–2019), including new parameters (red zone offense and defense) not found in the original study, machine learning techniques (logistic regression and decision tree analysis) are used to discover whether a coach should alter their end-of-game strategy based on the anticipated likelihood of winning the game should it go to overtime. For instance, if a coach is faced with a low likelihood of winning a game in overtime, calling more risky plays to try to win the game in regulation might increase their chance of winning. As coaches are invariant in their choice of the “defense first” coin toss decision, altering the decision lens to considering this end of game decision is a more relevant and impactful problem worthy of overtime game analysis.

## Data/Methodology

A variety of online websites were used to collect relevant game data. The NCAA.com website hosts a list of OT games played over the past 7 years (it was cross-checked with other online sources such as ESPN.com to insure accuracy). Additionally, the data reflecting a team's offensive and defensive red zone performance (explained in more detail below) was also found at the NCAA website. This data is only available for the past 7 years, thus the 7 years convenience data set.

Each overtime game was coded from the perspective of the team that chose the conventional wisdom strategy of “defense first,” determined either from the ESPN.com box score or, when it was not clear, from a search of newspaper articles describing the game itself. WIN is the 0/1 dependent variable, representing whether the defense first team won the game.

The home team (HOME) (game location) was noted in the game record (1 = home, 0 = visitor), as was the “point spread” (PSPREAD) of the game, each of the team's offensive and defensive red zone performance (four parameters in all, RZO, RZD, ORZO, ORZD, acronym clarified below), and the number of overtimes (OT) required.

Goldsheet.com was the main source of the game's point spread. This value indicates which team is favored to win (and by how many points) and is set by Las Vegas gambling casinos in attempt to level betting amounts on each team. As such, the point spread represents a surrogate measure of which team is “better” [and favored to win, see Smith and Capron (2018) for a more detailed discussion]. While the point spread may include other factors (theoretic home field advantage, bettor bias, among other things), it has long been used in football prediction research as a valid measure for team strength.

One of the seminal past works using point spread data in football game analysis was Stern (1991). In essence, he found empirically that the likelihood of a team winning a game is found by the cumulative normal distribution with a value of the point spread, the mean of 0, and a standard deviation of 14. Thus, a seven-point favorite would have a *Z* = (7–0)/14 or *Z*(0.5) or 69.14% change of winning, and a seven-point underdog would have a *Z*(−0.5) or 30.86% chance of winning. This study chose to transform the point spread data to the likelihood of winning based on this research (NORM).

The “red zone” is a football term referring to a team having possession inside the opponent 20-yard line (close to the end zone). The NCAA keeps track and ranks teams based upon the percentage of times they score in the red zone, including the number of touchdowns (six points) and field goals (three points). Curiously (and inaccurately), they rank the teams based on their percentage of scoring instead of the more insightful expected number of points a team scores in the red zone. Thus, the NCAA data was transformed by taking touchdowns × 7 (points plus assuming an extra point) + field goals × 3, then dividing by the number of times the team was in the red zone. This “expected value” is a more accurate representation of team red zone performance. The defensive red zone performance was similarly calculated for each team, reflecting the expected (or average) number of points scored when opponents crossed their 20-yard line.

The red zone data was coded from the perspective of the team that chose to go on defense first. RZO (Red Zone offense) and RZD (Red Zone defense) were the expected values for the “defense first team,” while ORZO and ORZD (O = Opponents) represented the same expected value measurement for the opponent team. As overtime rules gives each team possession of the ball at the 25 yards, just five yards greater than where the hypothetical red zone begins, it is hypothesized that a team's performance measured by RZO may be relevant to the outcome.

While the number of overtime periods necessary to reach the final game outcome was also procured and reported, the decision framework treats overtime as an “opaque box.” In essence, the model is trying to predict winners regardless of the process needed to achieve victory (i.e., the number of periods). The implications of investigating this process may be worthy of future research.

In a few cases, data was missing regarding team red zone performances (normally when non-division I-A teams were involved in an overtime game). In such a case, the average value for Red Zone performance was used in that record (~4.9 points per red zone trip).

A total of 243 usable data points (games) were identified. As the goal of the study was to discover actionable rules for the coaches, all games were used in creating the machine learning models (i.e., the training set). In the spirit of the challenges of creating “explainable Artificial intelligence” (XAI), some form of actionable and understandable set of “rules” or numerical assessments were desired that could be utilized by the decision-makers (coaches). This drove the choice of machine learning techniques (those that could be quantified and/or explained) as well as use of one set of data to create the models [e.g., Miller (2018)].

Given our goal of interpretable, implementable, and understandable results, two machine learning techniques were employed—logistic regression and SAS Enterprise Miner decision tree algorithm. Logistic regression would provide a probability on likelihood for teams to win games in overtime, while a decision tree approach creates a set of rules that could mimic a decision approach to help a coach decide how to tactically alter their end-of-game strategies. The output of both approaches will be contrasted in the following sections.

In both cases, the default parameters of SAS were used to generate the models/analysis. The logistic regression model (HPLOGISTIC) was used with Newton–Raphson with ridging for the optimization technique. Backward stepwise regression was used to limit the results to only significant variables in order to achieve better “explainable” solutions. For the decision tree analysis, all default parameters were used in the Enterprise Miner. This included the use of chi-squared measure for nominal splits and the entropy criteria for ordinal splits. Generic use of the tools would lead to greater ability for replication.

## Results

The following tables provide some of the relevant and interesting descriptive statistics of our 243-game database. Table 1 provides basic descriptive information regarding mean, standard deviation, and range.

**Table 1:** Descriptive statistics.

	**AVG**	**SD**	**MIN**	**MAX**
WIN	0.51	0.501	0	1
HOME	0.486	0.501	0	1
PSPREAD	−0.815	10.486	−41	34
OT	1.642	1.04	1	7
RZO	4.947	0.427	3.902	5.984
RZD	4.852	0.465	3.454	6.302
ORZO	4.971	0.442	3.54	6.093
ORZD	4.888	0.483	3.675	6.302
NORM	0.484	0.232	0.002	0.999

Table 2 shows the win–loss records of the teams that chose to go on defense first, by year. Overall, 51% of the teams that went on defense first won in overtime. We can see that the data does not support the conventional wisdom held by many that there is a significant advantage to being on defense first. Additionally, Table 2 shows the win–loss records of home teams involved in the overtime games. As some games were played at neutral sites, the total number of games does not match the numbers in the defense first column. Note that home teams won ~50.6% of the overtime games.

**Table 2:** Win–loss records—defense first and home team.

	**Defense first**			**Home**		
	**Win**	**Loss**		**Win**	**Loss**	
2019	15	15		15	14	
2018	19	14		17	14	
2017	13	21		14	16	
2016	21	21		24	16	
2015	22	13		17	16	
2014	19	16		15	17	
2013	15	19		14	20	
	124	119	51.03%	116	113	50.66%

Table 3 shows the distribution of games by the number of overtime periods. Note that this study is treating overtime as an “opaque box” and not explicitly considering the process of overtime. 62% of the total overtime games were decided in the first overtime period. The number of games won by the team that went on defense first (in the first overtime as a reference point) is shown in this table as well.

**Table 3:** Results by number of overtimes.

**Overtimes**	**Defense first**	**Overtime games**
	**Wins**	**Total**
1	71	151
2	27	52
3	21	30
4	2	5
5	2	3
6	0	1
7	2	2

Observe how 70% of the games won in the third overtime were teams that were on defense first in that period (and also in the first period!). The third period of OT is where a team must go for a two-point conversion after scoring a touchdown. Thus, because of this unique phenomenon, a “process look” at overtime games might provide additionally insight in future research.

Next, the two machine learning technique results are presented—logistic regression and decision tree analysis. Clearly, neither approach resulted in an outstanding predictive model—which shows that the overtime process is “fair” in the sense that each team has a good chance to win. However, from a predictive standpoint, this makes this a difficult problem.

When using logistic regression, the use of an intercept term was considered. When a full model was forced, the intercept value was not significant. When the intercept value was suppressed, and a backward elimination strategy employed, three variables were significant—the normalized point spread (NORM), red zone offense (RZO), and opponent red zone offense (ORZO). As the descriptive data indicates little to no evidence on any advantage to go “defense first” (which would be indicated in the significance of the intercept value), logistic regression with no intercept was chosen as an appropriate model.

Table 4 shows information about the logistic regression coefficients. Table 5 shows the classification matrix—which classified at a respectable but not outstanding 59.6% overall. The overall model was significant based upon the Likelihood ratio test (*p* = 0.0025) and the rejection of the Hosmer and Lemeshow goodness-of-fit test (*p* > 0.6962).

**Table 4:** Logistic regression parameters.

	**Parameter Estimate**			
**Parameter**	**Estimate**	**SE**	**T-value**	***P*-value**
RZO	0.4664	0.2388	1.95	0.0508
ORZO	−0.5894	0.2293	−2.57	0.0102
Norm	1.39	0.5737	2.42	0.0154

**Table 5:** Classification matrix.

	**Predicted**		
**Actual**		**Win**	**Loss**
	Win	81	57
	Loss	43	62

In interpreting the coefficients, it is not surprising that the larger the perceived strength of the team (increased normalized point spread) and the most effective (larger) red zone offense expected value, the higher the likelihood of the model predicting a victory for the reference team. Also note the larger negative impact the opponent red zone offense has on the predicted odds. Game location and any defensive parameters were eliminated as insignificant variables in the stepwise regression.

Table 6 assists looking at a few examples to help illustrate additional interpretation of the coefficients. Consider that the average red zone offense expected number of points was almost exactly 5.0 across the data set. So, with both teams performing at that level, if a team was a seven-point favorite, they will have a projected probability of winning the game in overtime of 58.6%; if a 14-point favorite, a projected probability of winning of 63.5%, and a 21-point favorite, a 66.4% likelihood of victory.

**Table 6:** Example parameters and win likelihood.

**RZO**	**ORZO**	**NORM**	**Win%**
5	5	7	58.6
		14	63.5
		21	66.4
4.75	5.25	7	52
		14	57.2
		21	60
4.5	5.5	7	45.5
		14	50.7
		21	53.8

Now consider the situation where the reference team has a poorer red zone offense (4.75) than the opponent (5.25). Using the same 7-, 14-, and 21-point favorite scenarios, the likelihood of the reference team winning decreases to 52, 57.2, and 60%, respectively. As the difference in red zone performance becomes more enhanced (see the third scenario in Table 6−4.5 vs. 5.5), the likelihood of a victory decreases to <50% even if the team is favored (by 7). These scenarios are depicted in Table 6.

Note that as an opponent red zone offense becomes “stronger” in a matchup, the likelihood of winning for the favorite team diminishes by ~3.2 units per 0.25 point increase. In practice, a coach would know the historical performance of each team's offense in the red zone and would also be able to assess through point spreads or other means for the relative strength of the teams. Therefore, in real time, they could gain a reasonable estimate on the likelihood of winning a game in overtime based upon this output. This possible “in-game” tactical decision is illustrated in the Discussion section.

Finally, the results of the decision tree analysis (using built-in pruning used by SAS Enterprise Miner with default settings) showed a better predictive accuracy, but the resulting rules were not as “implementable” as the logistic regression approach.

The derived rules from the Enterprise Miner used only the normalized point spread and the red zone offense parameters. If the normalized point spread was <0.4574 (which means the team on defense first was not the favored team), the suggested outcome was “LOSS” (74/118 cases).

If the normalized point spread was ≥0.4574, then the red zone offense was relevant. If the red zone offense averaged ≥4.43 (much less than the overall data set average), then the model predicted a “WIN” (74/106 cases). If it was <4.43, then the model predicted a loss (13/19 cases).

Using domain knowledge in the spirit of XAI, these rules in general could be further simplified: If a team is favored and their red zone offense is not terrible (since 4.43 is fairly low compared to the average expected points), predict a win, otherwise, predict a loss. Thus, the decision tree results in less helpful “rules” than the logistic regression.

The predictive accuracy is shown in Table 7 (66.0%). While the classification performance was better than the logistic regression approach, the insight provided by the logistic regression seems more value added to the decision-maker. A coach can consider the relative strength of teams and the ability of each team in the red zone to score when contemplating late game actions designed to either force the game into overtime or attempt to win the game outright during regulation time. Two simple examples illustrate how these results could be used as such in the Discussion section.

**Table 7:** Classification accuracy—decision tree.

	**Predicted**		
**Actual**		**Win**	**Loss**
	Win	74	50
	Loss	32	87

## Discussion

The two machine learning approaches struggled to predict the outcome of overtime games, though the results were improved over pure chance. Consider though the practical reality of overtime games—after having 70–90 plays from scrimmage during the regulation portion of the game, the outcome of overtime could come down to as few (or even less) than six plays. Thus, game results appear much more “random” and, frankly, that is the allure of the college football overtime process—it is exciting for fans because of the more unknown or unpredictable results.

This study once again dispels that being on defense first provides some sort of remarkable advantage to the team that wins the coin toss. The knowledge of this should be comforting to teams that lose the coin flip. The college football world is waiting for the coaching “rebel” who, when faced with the coin toss decision, starts choosing offense first in overtime games.

It is interesting to note that perhaps it is time to consider other “conventional wisdom” strategies or in-game tactics that rely on the assumption of “going last” is best (like the “defense first” strategy)? Examples might include penalty kicks in soccer, the home team batting last in baseball, and perhaps other scenarios in various sports.

As mentioned in the introduction, the results of the machine learning approaches presented might be more helpful when teams/coaches are faced with end-of-game decisions. Consider a scenario when late in the (regulation) game, a team scores a touchdown and trails by one point after the touchdown. They are then faced (as they are after every touchdown) with choosing between kicking the one-point extra point (with an ~97% chance of success) and sending the game into overtime or trying to run/pass a two-point conversion and “winning” the game if successful. Note that historically, two-point attempts have a success rate around 45%.

Here are two recent examples (another convenience sample) that illustrate how this approach might be implemented in real time, using the results of this study.

### Example 1: 2016 Season—Oklahoma State vs. Texas Tech

Oklahoma State is favored by 10 points. Their red zone offense averages 5.14 pts, whereas Texas Tech's average 5.63 points.

Texas Tech scores late in the game to pull within one point. They elect to kick the extra point to try to send the game into overtime (in a strange twist of irony, they miss the normally almost “sure thing” extra point). What would the results of the study suggest that Texas Tech should do to maximize the likelihood of a victory? Using the logistic regression equation, if they make the extra point and the game goes into overtime, they have a likelihood of between 46.5 and 48.1% to win the game (depending on the reference point—defense or offense first). Comparing this to the 45% chance of successfully completing the two-point extra point, and considering the 97% likelihood of a successful one-pt extra point, Texas Tech made the right decision to try to send the game into overtime (likelihood of winning with the 1-pt = 0.97^*^0.465 = 0.451 on the low end, or 0.97^*^0.481 = 0.467 on the high end). Unfortunately, sometimes making the right decision still leads to an unfavorable outcome.

### Example 2: 2018 Season—Oklahoma State vs. Oklahoma

Oklahoma is favored by 21 points. Oklahoma's red zone offense averages 5.485 points, whereas Oklahoma State's averages 5.37 points.

Oklahoma State scores late in the game to pull within one point. They elect to try a two-point conversion to win the game in regulation instead of kicking the one-point extra point to send the game into overtime. They are unsuccessful and lose. Their coach gets a lot of criticism for going for two points.

Using the derived logistic regression equation, Oklahoma State had between a 33.2 and 34.5% likelihood of winning an overtime game. As stated before, the two-point conversion typically has a 45% success rate. Thus, Oklahoma State appeared to make the right end-of-game decision … even though they too lost!

There are likely other similar examples that one can analyze, and perhaps other scenarios where a team may give up the chance for a game-tying field goal toward the end of the game and tries to score a touchdown to prevent overtime. In summary, the most significant outcome of this study may be the additional insight provided on these end-of-game decisions.

## Conclusion

All studies have limitations. We have used the past 7 years of data. Seeing that the red zone offense plays a role in predicting outcomes, it will be difficult to go back in the game archives much further without very strenuous play-by-play assessment of hard-to-find game data. Perhaps there are surrogate measures one can use instead of red zone efficiency that can assist us in gaining further historical insight. It is clear from the analysis though that there is no advantage to being on defense first. There is an opportunity for a innovate coach to exploit this phenomenon.

Is there anything that other sports can glean from this study? It was previously mentioned that the “last bats” philosophy of baseball is what drives the conventional wisdom in college football. Does the data support this? Perhaps the biggest takeaway from the study to other sports is find data to question convention wisdom in terms of strategies. The “Moneyball” era in baseball questioned historical ways of building a team (Lewis, 2003). The same may be true at the micro level in a variety of sports. As an example, there is much work in big data and AI in soccer (Rein and Memmert, 2016).

The machine learning/prediction models did not predict with outstanding accuracy. Because college football overtime process is “fair” by competitive standards, this is not surprising. The models developed did provide some insight into predicting outcome and can inform the knowledgeable coach to consider the variables that influence possible overtime success as the end of game nears.

The process of overtime (gong from period to period) was not considered and is beyond the scope of this study. Thus, does something happen as a game moves from overtime one to overtime period two? Note the unique performance of teams in overtime period three (a high percentage of defense first teams win). This process may be worthy of future investigation.

College football is one sport where analytics can play a role in helping a team's success. This study looked at archival data and determined that there is some evidence for a team's red zone success (along with the relative strength of a team) to influence the likelihood of a win in overtime. Trends in overtime games should be followed in the future to see if this model holds up as the game continues to evolve and change over time.

## Data Availability Statement

The datasets generated for this study are available on request to the corresponding author.

## Author Contributions

The author confirms being the sole contributor of this work and has approved it for publication.

## Conflict of Interest

The author declares that the research was conducted in the absence of any commercial or financial relationships that could be construed as a potential conflict of interest.
